# Association Between the nt230(del4) Mutation and c.‐6‐180T>G Polymorphism in the Canine ABCB1 Gene

**DOI:** 10.1155/vmi/1075604

**Published:** 2026-07-11

**Authors:** Orsolya Palócz, Réka Wágner, György Csikó

**Affiliations:** ^1^ Department of Pharmacology and Toxicology, University of Veterinary Medicine, Budapest, Hungary, univet.hu

**Keywords:** ABCB1, deletion mutation, dog, MDR1, P-glycoprotein, SNP

## Abstract

A 4‐base pair deletion in the ABCB1 (MDR1) gene, resulting in a frameshift and truncated, nonfunctional P‐glycoprotein, is frequently observed in certain dog breeds and is known to cause drug sensitivity. Another variant, a single nucleotide substitution (c.‐6‐180T > G) located near the gene’s promoter region, has also been identified; however, its clinical significance remains unclear. This study aimed to determine the genotype distribution of the nt230(del4) deletion and the c.‐6‐180T > G substitution across various dog breeds and to evaluate whether an association exists between these variants. A total of 263 client‐owned dogs from 17 breeds were genotyped for both mutations. Genomic DNA was extracted using a spin column–based method. The deletion was detected using a modified allele‐specific real‐time PCR assay, and the SNP was assessed via PCR followed by RFLP analysis. The nt230(del4) mutation was identified exclusively in collie‐lineage breeds, including collie, Shetland sheepdog, Australian shepherd, bobtail, and white Swiss shepherd. The c.‐6‐180T > G SNP was widespread across all breeds, with a G allele frequency of 35%. Genotype distribution included 111 T/T homozygotes, 119 T/G heterozygotes, and 33 G/G homozygotes. Notably, all individuals carrying the deletion also possessed at least one G allele, suggesting a co‐occurrence between the two variants. The observed association between the nt230(del4) deletion and the c.‐6‐180T > G SNP in this cohort may reflect shared ancestry and/or local linkage disequilibrium. Further research is warranted to explore the functional consequences of this SNP and its potential role in canine drug response.

## 1. Introduction

One of the earliest identified members of the ATP‐binding cassette (ABC) transporter family is the P‐glycoprotein (P‐gp) efflux transporter encoded by the *ABCB1* gene, formerly called multidrug resistance 1 (MDR1) [1]. P‐gp (ABCB1) is expressed predominantly in the apical (luminal) membrane of epithelial and endothelial cells and plays an important role in limiting xenobiotic absorption, facilitating elimination into bile, urine, and the intestinal lumen, and protecting tissues from drug accumulation [2]. One of its most important physiological roles is maintaining the blood–brain barrier by restricting entry of xenobiotics into the central nervous system (CNS).

Drug sensitivity associated with altered ABCB1 function has long been recognized in collie dogs. The underlying mechanism became clearer after studies in *mdr1a* knockout mice demonstrated marked accumulation of ivermectin in the brain and increased neurotoxicity compared with wild‐type animals [3]. This observation led to the discovery of a 4‐base pair deletion in the canine ABCB1 gene [4]. The nt230 (del4) deletion results in a frameshift at amino acid position 75, followed by a premature stop codon at amino acid position 91, producing a severely truncated nonfunctional transporter [5]. Several dog breeds are predisposed to this mutation, with the highest mutant allele frequencies reported in collies and Shetland sheepdogs [6].

Dogs carrying the deletion may exhibit increased susceptibility to adverse drug reactions following the administration of, otherwise, routinely used drugs. Clinically relevant substrates include macrocyclic lactones (e.g., ivermectin), opioid and sedative agents (e.g., butorphanol and acepromazine), antidiarrheal drugs (e.g., loperamide), immunosuppressants (e.g., cyclosporine), and certain chemotherapeutic agents (e.g., vincristine). Clinical manifestations may include ataxia, lethargy, neurological signs, and severe intoxication. Dogs homozygous for the deletion show increased sensitivity to certain P‐gp substrates although heterozygotes might show intermediate susceptibility [7].

In addition to the deletion mutation, a polymorphic thymine‐to‐guanine substitution located near the promoter region of the canine ABCB1 gene (c.‐6‐180T > G) has previously been described [8]. Because promoter regions may influence transcriptional regulation, this polymorphism has attracted interest as a potential modifier of ABCB1 expression. Among the numerous reported canine ABCB1 variants, c.‐6‐180T > G was selected for the present study because it represents one of the few polymorphisms previously investigated for possible pharmacological relevance and has been discussed in relation to phenobarbital responsiveness in dogs with idiopathic epilepsy [8, 9]. However, its functional and clinical significance remains uncertain.

The aim of our study was to determine the genotype distribution of both the nt230(del4) mutation and the c.‐6‐180T > G substitution across predisposed and nonpredisposed dog breeds and evaluate whether an association or co‐occurrence pattern exists between these variants.

## 2. Materials and Methods

Blood samples were collected from 263 client‐owned dogs; the owners gave signed informed consent for study enrollment. The dogs showed no apparent clinical signs at enrollment. The tested dog breeds were German shepherd (61), Hungarian pointer (47), Shetland sheepdog (36), border collie (35), white Swiss shepherd (17), collie (17), mudi (14), Hungarian greyhound (14), Australian shepherd (7), puli (4), dachshund (3), old English sheepdog (1), golden retriever (1), Labrador retriever (2), shar pei (1), fox terrier (1), whippet (1), and Yorkshire terrier (1). Genomic DNA was purified from all blood samples via the column‐based method. Genomic DNA was diluted to 2 ng/μL concentration prior to PCR analysis. The nt230(del4) mutation was determined by allele‐specific polymerase chain reaction modified from Baars et al. [10]. The following primers were used: forward wild type (Wt): 5′‐TTGGAAACATGACAGATAGC‐3′, forward deletion (Del): 5′‐CGTTTTTGGAAACATGACAGC‐3′ and reverse 5′‐AACTTCCTGGGATCTTTCTG‐3′. For each sample, two independent PCR reactions were performed using primer pairs specific to the wild type and the deletion alleles.

The ABCB1 c.‐6‐180T > G polymorphism was determined by the PCR‐RFLP method according to Mizukami et al. [8]. The applied primers were: forward (5′‐GCAGTGGGGTGAGAACTAGA‐3′) and reverse (5′‐CGCAAGCCATGTAAGGTATG‐3′). PCR analyses for both assays were performed using the iQ SYBR Green Supermix kit (Bio‐Rad, Hercules, CA, USA) on a CFX Opus Real‐Time PCR System (Bio‐Rad). Thermal cycling conditions were identical for both assays and consisted of an initial denaturation at 95°C for 3 min followed by 30 cycles of 95°C for 15 s, 60°C for 30 s, and 72°C for 30 s. Fluorescence acquisition was performed for 10 s at the end of each cycle.

### 2.1. Statistical Analysis

Statistical analyses were performed using R Version 4.4.3 (R Core Team, 2025). Association between ABCB1 c.‐6‐180T > G genotype (T/T, T/G, G/G) and nt230(del4) deletion status (wild type, heterozygous carrier, and homozygous deletion carrier) was evaluated using a chi‐square test of independence. Effect size was quantified using Cramér’s V. Statistical significance was set at *α* = 0.05, and all tests were two‐sided.

## 3. Results

All 263 dogs were tested for both the deletion and the substitution; the raw data are supplied in Table S1. The nt230(del4) deletion was present in collie, Shetland sheepdog, Australian shepherd, old English sheepdog, and white Swiss shepherd dogs, and none of the other investigated breeds were carrying the *ABCB1* deletion. Altogether, among 78 predisposed dogs, without the border collie breed, 44 carried the 4‐bp deletion of ABCB1 in one copy and 17 carried in two copies. Table 1 summarizes the genotype distribution of the two tested genetic markers for breeds with the largest sample representation.

**TABLE 1:** Distribution of the nt230(del4) and the c.‐6‐180T > G SNP of canine ABCB1 gene within the breeds with the highest number of individuals.

Breed	Wild type	Heterozygous deletion	Homozygous deletion	T/T	T/G	G/G	*n*
German shepherd	61	0	0	36	23	2	61
Hungarian pointer (Magyar vizsla)	47	0	0	22	24	1	47
Hungarian greyhound (Magyar agar)	14	0	0	5	8	1	14
Mudi	14	0	0	10	4	0	14
Shetland sheepdog	8	18	10	3	17	16	36
Border collie	35	0	0	19	14	2	35
White Swiss shepherd	15	2	0	4	9	4	17
Collie	9	4	4	8	5	4	17

**FIGURE 1:**
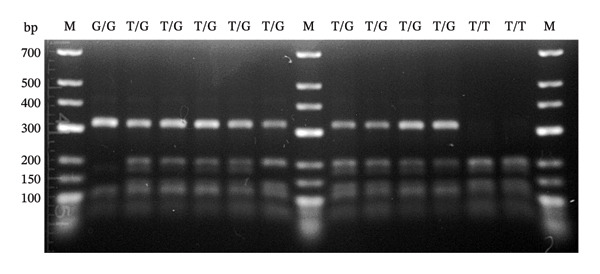
Representative electrophoretogram of agarose gel after RFLP assay. The amplified DNA digested with restriction endonuclease MboI. Homozygous wild type (T/T), heterozygote (T/G), homozygous mutant type (G/G), molecular size markers (M), and base pair (bp).

In the G/G homozygous dog, the amplified DNA segment was digested into 2 fragments (313 and 103 bp bands); in the T/T homozygous wild‐type dog, the amplified band was digested into 3 fragments (191, 122, and 103 bp bands); and in the T/G heterozygous dog, it was digested into 4 fragments (313, 191, 122, and 103 bp bands) (Figure 1). The G allele was present in all dog breeds with highest abundance in homozygote form in Shetland sheepdogs (Table 1). Among all samples, the homozygote T/T wild type was present in 111 dogs, the heterozygote T/G was detected in 119 individuals, and 33 dogs carried the homozygote G/G genotype. The G allele frequency was 35% in the investigated population.

All dogs that carried the nt230(del4) deletion in at least one copy always carried the G allele in at least one copy. All 45 dogs homozygous for the deletion carried the G allele, 22 of them in two copies and 23 of them in one copy. Among the 218 dogs without the deletion, 107 carried the G allele, and only 11 of them in two copies. A chi‐square test of independence demonstrated a statistically significant association between the deletion status and c.‐6‐180T > G genotype (*χ* ^2^(4) = 89.72, *p* < 0.001, Cramér’s V = 0.41), indicating a moderate‐to‐strong association within this cohort.

## 4. Discussion

Our findings demonstrate an observed association and consistent co‐occurrence between two genetic alterations in the canine ABCB1 gene: the nt230(del4) deletion and the c.‐6‐180T > G single nucleotide polymorphism (SNP). Notably, all dogs carrying the deletion also possessed at least one copy of the G allele at the SNP site. This consistent co‐occurrence may suggest potential linkage disequilibrium between the two variants, likely due to their close proximity on the genome and shared breed ancestry. Similar patterns of coinheritance have been observed in genetically related dog populations [11, 12].

The G allele was especially prevalent in Shetland Sheepdogs, with 92% of individuals carrying at least one copy, compared to 52% in other breeds combined. Published data regarding the distribution of the c.‐6‐180T > G SNP in collie‐lineage breeds outside Hungary remain limited and are largely restricted to border collies. In Japan, Mizukami et al. [8] reported a G allele frequency of 24.9% among 472 border collies, while Alves et al. [13] observed a frequency of 24% in 236 border collies from Switzerland and Germany. Similarly, in our cohort, the G allele frequency in border collies was 26%, suggesting comparable distribution across geographically distinct populations.

Consistent with earlier reports, the nt230(del4) mutation was absent in our border collie cohort, despite being present in other collie‐lineage breeds [14].

Among Hungarian dog breeds, we found no individuals carrying the deletion mutation, even though we sampled 79 dogs—the largest such dataset reported to date. The presence of the deletion in Hungarian dog breeds is not reported in the scientific literature, these breeds are rarely tested for the deletion [1, 15] probably because there is no reported drug adverse effect in connection with the nt230(del4) in Hungarian breeds although larger studies would be required before excluding the presence of rare alleles in these populations. However, more than half of these individuals carried the G allele, indicating that the c.‐6‐180T > G polymorphism may be more widely distributed in the general dog population than the deletion mutation.

While the clinical significance of the deletion mutation is well established—particularly regarding increased sensitivity to drugs like ivermectin and certain chemotherapeutics—the functional relevance of the c.‐6‐180T > G SNP remains unclear. It has been hypothesized that this substitution might alter promoter activity and increase ABCB1 expression [8], potentially affecting drug bioavailability and resistance. However, direct experimental confirmation is lacking. Future studies should, therefore, investigate the functional impact of this polymorphism using promoter activity assays, quantitative analysis of ABCB1 mRNA and P‐gp expression, and pharmacogenetic studies evaluating whether c.‐6‐180T > G influences drug disposition or treatment response in dogs. Interestingly, this SNP has been associated with phenobarbital resistance in dogs with idiopathic epilepsy although results across studies have not been fully consistent [8, 9, 13]. These inconsistencies may reflect differences in breed composition, sample size, clinical endpoints, treatment protocols, and the possible contribution of additional regulatory or epigenetic mechanisms affecting ABCB1 expression. Furthermore, if the proposed increase in P‐gp expression is confirmed experimentally, altered tissue drug exposure could theoretically contribute to interindividual differences in drug response; however, this hypothesis remains speculative and requires functional validation [16].

In other species, including humans, certain ABCB1 polymorphisms have been shown to influence alternative splicing, drug response, and disease susceptibility [17, 18]. It therefore remains plausible that the c.‐6‐180T > G variant interacts with other regulatory mutations to modulate P‐gp expression or function in dogs; however, current evidence remains preliminary.

This study contributes to the growing body of evidence on the genetic variability of ABCB1 in dogs, particularly by documenting the distribution of the c.‐6‐180T > G SNP across diverse breeds and demonstrating consistent co‐occurrence with the nt230(del4) deletion in our population. Further research is warranted to explore the mechanistic role of this SNP in ABCB1 gene regulation and its potential impact on drug pharmacokinetics and therapeutic outcomes.

The present study has several limitations. Breed representation was uneven, and some breeds were represented by small sample numbers, limiting population‐level inference. The cohort was not designed for population genetic analysis and functional validation of c.‐6‐180T > G was beyond the scope of this work. Therefore, the observed association should not be interpreted as evidence of causality or universal linkage across dog populations.

## 5. Conclusions

In conclusion, we identified an association between the ABCB1 nt230(del4) deletion and the c.‐6‐180T > G SNP in dogs, with both variants showing consistent co‐occurrence among deletion carriers within this cohort. All dogs carrying the nt230(del4) deletion also harbored at least one G allele at the c.‐6‐180T > G locus, a pattern that may reflect shared ancestry and/or local linkage disequilibrium. While the deletion mutation was restricted to collie‐related breeds, the SNP was more widely distributed across all tested breeds. The observed association warrants further investigation to clarify possible functional consequences and its potential relevance to veterinary pharmacogenetics.

## Funding

This study was supported by the 12190/2017/FEKUTSTRAT grant of the Hungarian Ministry of Human Resources and the European Union and cofinanced by the ÚNKP‐21‐2 New National Excellence Program of the Ministry for Innovation and Technology from the source of the National Research, Development and Innovation Fund.

## Conflicts of Interest

The authors declare no conflicts of interest.

## Supporting Information

Additional supporting information can be found online in the Supporting Information section.

## Supporting information


**Supporting Information** Table S1. Supporting table of the research data listing all individuals involved in the study, containing the deletion and substitution results of all dogs. “Wild” refers to wild‐type dogs that do not carry a deletion of ABCB1, “carrier” refers to heterozygous deletion carriers, and “homozygous deletion carrier” refers to dogs carrying two copies of nt230(del4).

## Data Availability

The data that support the findings of this study are available in this paper and the supporting information of this article.

## References

[1] Gramer I. , Leidolf R. , Döring B. et al., Breed Distribution of the nt230(del4) MDR1 Mutation in Dogs, The Veterinary Journal. (2011) 189, no. 1, 67–71, 10.1016/j.tvjl.2010.06.012. 20655253

[2] Geyer J. , Döring B. , Godoy J. R. , Leidolf R. , Moritz A. , and Petzinger E. , Frequency of the nt230 (Del4) MDR1 Mutation in Collies and Related Dog Breeds in Germany, Journal of Veterinary Pharmacology and Therapeutics. (2005) 28, no. 6, 545–551, 10.1111/j.1365-2885.2005.00692.x. 16343287

[3] Schinkel A. H. , Smit J. J. , van Tellingen O. et al., Disruption of the Mouse mdr1a P-glycoprotein Gene Leads to a Deficiency in the blood-brain Barrier and to Increased Sensitivity to Drugs, Cell. (1994) 77, no. 4, 491–502, 10.1016/0092-8674(94)90212-7. 7910522

[4] Mealey K. L. , Bentjen S. A. , Gay J. M. , and Cantor G. H. , Ivermectin Sensitivity in Collies is Associated With a Deletion Mutation of the mdr1 Gene, Pharmacogenetics. (2001) 11, no. 8, 727–733, 10.1097/00008571-200111000-00012. 11692082

[5] Geyer J. and Janko C. , Treatment of MDR1 Mutant Dogs With Macrocyclic Lactones, Current Pharmaceutical Biotechnology. (2012) 13, no. 6, 969–986, 10.2174/138920112800399301. 22039792 PMC3419875

[6] Palocz O. , Major K. B. , Csigo B. et al., Prevalence of ABCB1/MDR1 Gene Mutation in Certain Hungarian Canine Population, Magyar Allatorvosok Lapja. (2018) 140, no. 8, 495–500.

[7] Beckers E. , Casselman I. , Soudant E. et al., The Prevalence of the ABCB1-1Δ Variant in a Clinical Veterinary Setting: The Risk of Not Genotyping, PLoS One. (2022) 17, no. 8, 10.1371/journal.pone.0273706. PMC9423603 36037240

[8] Mizukami K. , Yabuki A. , Chang H. S. et al., High Frequency of a Single Nucleotide Substitution (c.-6-180T>G) of the Canine MDR1/ABCB1 Gene Associated With Phenobarbital-Resistant Idiopathic Epilepsy in Border Collie Dogs, Disease Markers. (2013) 35, no. 6, 669–672, 10.1155/2013/695918. 24302812 PMC3834651

[9] Gagliardo T. , Gandini G. , Gallucci A. et al., ABCB1 c.-6-180T>G Polymorphism and Clinical Risk Factors in a Multi-Breed Cohort of Dogs With Refractory Idiopathic Epilepsy, The Veterinary Journal. (2019) 253, 10.1016/j.tvjl.2019.105378. 31685133

[10] Baars C. , Leeb T. , von Klopmann T. , Tipold A. , and Potschka H. , Allele-Specific Polymerase Chain Reaction Diagnostic Test for the Functional MDR1 Polymorphism in Dogs, The Veterinary Journal. (2008) 177, no. 3, 394–397, 10.1016/j.tvjl.2007.05.020. 17644437

[11] Neff M. W. , Robertson K. R. , Wong A. K. et al., Breed Distribution and History of Canine mdr1-1Delta, a Pharmacogenetic Mutation that Marks the Emergence of Breeds From the Collie Lineage, Proceedings of the National Academy of Sciences of the United States of America. (2004) 101, no. 32, 11725–11730, 10.1073/pnas.0402374101. 15289602 PMC511012

[12] Geyer J. , Klintzsch S. , Meerkamp K. et al., Detection of the nt230(del4) MDR1 Mutation in White Swiss Shepherd Dogs: Case Reports of Doramectin Toxicosis, Breed Predisposition, and Microsatellite Analysis, Journal of Veterinary Pharmacology and Therapeutics. (2007) 30, no. 5, 482–485, 10.1111/j.1365-2885.2007.00885.x. 17803743

[13] Alves L. , Hülsmeyer V. , Jaggy A. , Fischer A. , Leeb T. , and Drögemüller M. , Polymorphisms in the ABCB1 Gene in Phenobarbital Responsive and Resistant Idiopathic Epileptic Border Collies, Journal of Veterinary Internal Medicine. (2011) 25, no. 3, 484–489, 10.1111/j.1939-1676.2011.0718.x. 21488961

[14] Firdova Z. , Turnova E. , Bielikova M. , Turna J. , and Dudas A. , The Prevalence of ABCB1:c.227_230delATAG Mutation in Affected Dog Breeds From European Countries, Research in Veterinary Science. (2016) 106, 89–92, 10.1016/j.rvsc.2016.03.016. 27234542

[15] Marelli S. P. , Polli M. , Frattini S. , Cortellari M. , Rizzi R. , and Crepaldi P. , Genotypic and Allelic Frequencies of MDR1 Gene in Dogs in Italy, Vet Rec Open. (2020) 7, no. 1, 10.1136/vetreco-2019-000375. PMC7319724 32617164

[16] Lee J. J. , Lin H. Y. , Chen C. A. , Lin C. S. , and Wang L. C. , Development of an Oligonucleotide Microarray for Simultaneous Detection of Two Canine MDR1 Genotypes and Association Between Genotypes and Chemotherapy Side Effects, Journal of Veterinary Science. (2019) 20, no. 1, 27–33, 10.4142/jvs.2019.20.1.27. 30481983 PMC6351760

[17] Zhang S. , Wang J. , Zhang A. et al., A SNP Involved in Alternative Splicing of ABCB1 is Associated With Clopidogrel Resistance in Coronary Heart Disease in Chinese Population, Aging (Albany NY). (2020) 12, no. 24, 25684–25699, 10.18632/aging.104177. 33232268 PMC7803500

[18] Maués T. , El-Jaick K. B. , Costa F. B. et al., Could Polymorphisms in ABCB1 Gene Represent a Genetic Risk Factor for the Development of Mammary Tumors in Dogs?, The Veterinary Journal. (2019) 248, 58–63, 10.1016/j.tvjl.2019.04.010. 31113564

